# Mechanistic Study of Bakuchiol-Induced Anti-breast Cancer Stem Cell and *in Vivo* Anti-metastasis Effects

**DOI:** 10.3389/fphar.2017.00746

**Published:** 2017-10-18

**Authors:** Li Li, Chi C. Liu, Xueping Chen, Shisan Xu, Sinai Hernandez Cortes-Manno, Shuk H. Cheng

**Affiliations:** ^1^Department of Biomedical Sciences, City University of Hong Kong, Kowloon, Hong Kong; ^2^Vitargent (International) Biotechnology Limited, Sha Tin, Hong Kong; ^3^Department of Infectious Diseases and Public Health, City University of Hong Kong, Kowloon, Hong Kong; ^4^Department of Physics, City University of Hong Kong, Kowloon, Hong Kong

**Keywords:** bakuchiol, breast cancer stem cells, zebrafish xenografts, apoptosis, metastasis

## Abstract

Cancer stem cells are involved in cancer establishment, progression, and resistance to current treatments. We demonstrated the *in vitro* and *in vivo* anti-breast cancer effect of bakuchiol in a previous study. However, the ability of bakuchiol to target breast cancer stem cells (BCSCs) and inhibit breast cancer metastasis remains unknown. In the current study, we used the cell surface markers CD44 and CD24 to distinguish BCSCs from MCF-7 cells. Bakuchiol inhibited mammosphere formation and aldehyde dehydrogenase activity in BCSCs. Moreover, bakuchiol induced apoptosis and suppressed the mitochondrial membrane potential of BCSCs. Bakuchiol upregulated the expression levels of pro-apoptotic genes, BNIP3 and DAPK2. Bakuchiol induced oxidative stress and altered lipogenesis in BCSCs. In zebrafish xenografts, bakuchiol inhibited breast cancer cell metastasis *in vivo.* In addition, bakuchiol altered the expression levels of metastasis-related genes through upregulating CK18 and downregulating Notch3, FASN, TGFBR1, and ACVR1B. Our study provides evidence for the anti-breast cancer potential of bakuchiol.

## Introduction

Breast cancer is the second leading cause of cancer death among the women in the United States. Both metastases found at diagnosis (*de novo*) and those occurring later (recurrence) are considered the most severe forms of breast cancer (Mariotto et al., 2017). The 5-year survival rate of stage 4 metastatic breast cancer is 26%, which is much lower than the survival rates of the early stage breast cancers^1^. Tumors contain heterogeneous cell populations, in which a small subpopulation of cells, known as cancer stem cells, is responsible for tumor initiation, maintenance, metastasis, and recurrence (Velasco-Velázquez et al., 2011; Shiozawa et al., 2013; Peitzsch et al., 2017). Tumorigenesis can be induced in immunodeficient mice through the injection of as few as 200 breast cancer stem cells (BCSCs), which are identified on the basis of the expression of the cell surface makers, such as CD44^+^/CD24^-/low^ (Al-Hajj et al., 2003). Traditional cancer therapies are effective at debulking certain tumors but often fail to produce long-term clinical remissions given their inability to eradicate the BCSCs population. Thus, therapies that target BCSCs may improve the recurrent and metastatic rates.

Phytoestrogens are plant-derived substances that are structurally similar to the mammalian steroid hormone E2. Although the anti-cancer effects of phytoestrogens have been widely investigated (reviewed by Patisaul and Jefferson, 2010; Bilal et al., 2014), few studies have focused on the effects of phytoestrogens on BCSCs. Soy isoflavone genistein and blueberry polyphenolic acids repress mammosphere formation (Montales et al., 2012, 2013). Resveratrol inhibits BCSCs through the suppression of the Wnt/β-catenin and lipogenesis signaling pathway (Pandey et al., 2011; Fu et al., 2014). *In vitro*, *in vivo*, and clinical studies on the effects of phytoestrogen on metastasis have mainly focused on genistein, daidzein, and resveratrol. The anti-metastatic effects of these compounds have been reported in several tumor models, such as prostate cancer, breast cancer, melanoma, liver cancer, and colorectal cancer (Menon et al., 1998; Vantyghem et al., 2005; Gu et al., 2009; Sheth et al., 2012; Pavese et al., 2014; Ji et al., 2015).

Bakuchiol is a meroterpene found in the traditional Chinese herbal medicine *Fructus Psoraleae*, the dried ripe fruit of *Psoralea corylifolia L.* (Fabaceae). Bakuchiol possesses estrogenic, anti-microbial, anti-inflammatory, anti-oxidative, anti-osteoporosis, anti-depression, or anti-stress, and anti-cancer activities (reviewed by Li et al., 2016). In a previous study, we demonstrated the *in vitro* and *in vivo* inhibitory effect of bakuchiol on breast cancer (Li et al., 2016). However, the effects of bakuchiol on the growth of BCSCs and breast cancer metastasis have yet to be reported. We designed the current study according to the doses used in our previous *in vitro* and *in vivo* experiments (Li et al., 2016). The elucidation of pathways that how bakuchiol regulates BCSCs may lead to the identification of potential therapeutic targets. Prospective results will provide important evidence for the potential of bakuchiol as an anti-breast cancer agent.

## Materials and Methods

### Cell Culture

MCF-7 (ATCC: HTB-22) were purchased from American Type Culture Collection (ATCC, Manassas, VA, United States) and routinely maintained in Dulbecco’s modified Eagle’s medium (DMEM) supplemented with 10% fetal bovine serum (FBS), 0.37% Na_2_CO_3_, 50 units/mL penicillin, and 50 μg/mL streptomycin. The cells were incubated at 37°C in a humidified atmosphere with 5% CO_2_.

### Effects of Bakuchiol on Mammosphere Formation in MCF-7 Cells

Complete MammoCult medium was freshly prepared with 45 mL MammoCult basal medium (Stemcell Technologies, Canada), 5 mL growth supplement (Stemcell Technologies, Canada), 200 μL heparin adjusted to a final concentration of 4 μg/mL (Stemcell Technologies, Canada), 250 μL hydrocortisone adjusted to a final concentration of 0.48 μg/mL (Sigma, St. Louis, MO, United States), and 500 μL 100× penicillin and streptomycin (Invitrogen, Carlsbad, CA, United States). Complete MammoCult medium was filter-sterilized with a 0.22-μm syringe filter.

MCF-7 cells were collected and seeded in ultra-low attachment six-well plates (BD Biosciences, San Jose, CA, United States) with complete MammoCult medium at a density of 20,000 cells/well. Bakuchiol {4-[(1E,3S)-3-ethenyl-3,7-dimethylocta-1,6-dienyl] phenol} purity 98% by HPLC was obtained from Enzo (Farmingdale, NY, United States). Cells were exposed to ethanol (vehicle control, 0.1% v/v, same amount in all the treatment conditions) or treated with 4 or 7 μg/mL bakuchiol, then allowed to grow for a week to form mammospheres (p1). Mammospheres from each group were collected and trypsinized separately. Cells from different groups were seeded and treated with ethanol or bakuchiol again for a week to form mammospheres (p2). Cell images were taken under an inverted microscope (model DMI3000 B, Leica Microsystems, Germany).

### Cell Sorting for the Identification of CD44^+^/CD24^-/low^ Populations from MCF-7 Cells

Cells were collected and washed with staining buffer (PBS containing 1% BSA). Then, 1 × 10^6^ cells were resuspended in 100 μL staining medium with 20 μL fluorochrome-conjugated antibodies for 30 min on ice. Cells were washed with staining buffer and filtered using a strainer cap tube (BD Bioscience, San Jose, CA, United States) to avoid cell clumps.

Cell phenotypes were determined by flow cytometry using a FACS Aria III cell-sorting flow cytometer (BD Biosciences, San Jose, CA, United States) with FACS Diva 6.1 software (BD Biosciences, San Jose, CA, United States). Several dot-plots of different parameters were created on a global worksheet. Then, gates were drawn on corresponding populations of breast cancer cells to identify the subpopulation of interest, including SSC-A versus FSC-A for live cells, SSC-A versus PE-A for CD24^+^ cells, SSC-A versus FITC-A for CD44^+^ cells, and PE-A versus FITC-A for CD44^+^/CD24^-/low^ cells. The CD44^+^/CD24^-/low^ cells were BCSCs.

### Effect of Bakuchiol on Mammosphere Formation in BCSCs

A total of 1 × 10^6^ CD44^+^/CD24^-/low^ cells were seeded in a low-attachment, 60 mm plate in complete MammoCult medium with ethanol (vehicle control) or different bakuchiol concentrations. Cells were allowed to grow for 7 days to form mammospheres. Mammospheres were imaged at 8–10 random fields on days 4 and 7 with a phase contrast microscope.

### Determination of ALDH Activity

CD44^+^/CD24^-/low^ cells in low-attachment plates were exposed to ethanol (vehicle control) or different bakuchiol concentrations for 4 days. Aldehyde dehydrogenase (ALDH) activity was determined by using ALDEFLUOR Kit (Stemcell Technologies, Canada) according to manufacturer’s instructions. Briefly speaking, cells were incubated in ALDEFLUOR assay buffer containing ALDH substrate. In each treatment condition a sample of cells was stained under identical conditions with diethylaminobenzaldehyde (DEAB), a specific ALDH inhibitor, as negative control. Following incubation, the cells were collected, resuspended in ALDEFLUOR assay buffer, and analyzed with flow cytometry (Becton Dickinson, Mountain View, CA, United States).

### Annexin-V/PI Staining for Apoptosis Analysis

CD44^+^/CD24^-/low^ cells in low-attachment plates were exposed to ethanol (vehicle control) or different bakuchiol concentrations for 4 days. Annexin-V/propidium iodide (PI) (Promega, Fitchburg, WI, United States) staining was conducted as described by the manufacturer.

### Mitochondrial Membrane Potential Analysis

CD44^+^/CD24^-/low^ cells in low-attachment plates were exposed to ethanol (vehicle control) or different bakuchiol concentrations for 4 days. JC-1 staining (Invitrogen, Carlsbad, CA, United States) was conducted in accordance with the manufacturer’s instructions.

### ROS Production Analysis

The production of reactive oxygen species (ROS) was determined by using a ROS/superoxide detection kit (Enzo, Farmingdale, NY, United States). CD44^+^/CD24^-/low^ cells were exposed to ethanol (vehicle control) or different bakuchiol concentrations for 4 days. In accordance with the manufacturer’s instructions, staining buffer with oxidative stress detection reagent (green fluorescence) and superoxide detection reagent (orange fluorescence) were added to washed, trypsinized, and resuspended cells. The stained cells were analyzed by flow cytometry (Becton Dickinson, Mountain View, CA, United States). The ROS inhibitor (*N*-acetyl-L-cysteine) was used as negative control.

### Lipid Content Determination

Intracellular lipid content was quantified using AdipoRed Assay Reagent (Lonza, Walkersville, MD, United States). AdipoRed is a solution of the hydrophilic stain Nile Red, which can stain intracellular lipid droplets. CD44^+^/CD24^-/low^ cells were treated with ethanol (vehicle control) or different bakuchiol concentrations for 4 days. Cells were collected, washed, and stained with AdipoRed in accordance with the manufacturer’s recommendations. After 10 min of staining, fluorescence was measured using a SpectraMax Microplate Reader (Molecular Devices, Hong Kong) under excitation at 485 nm and emission at 572 nm.

### Semi-quantitative Real-Time PCR

After CD44^+^/CD24^-/low^ cells were exposed to ethanol (vehicle control) or different bakuchiol concentrations for 4 days, RNA extraction, genomic DNA decontamination, and semi-quantitative real-time PCR were performed as described in our previous study (Li et al., 2016). Primers are listed in **Table 1**.

**Table 1:** Primers for quantitative real-time PCR

Gene	Forward (5′–3′)	Reverse (5′–3′)
GAPDH	TCCCTGAGCTGAACGGGAAG	GGAGGAGTGGGTGTCGCTGT
BNIP3	CCTGGGTAGAACTGCACTTCAGCAAT	TTCATGACGCTCGTGTTCCTCATGCT
DAPK2	CTTTGATCTCAAGCCAGAAAACATT	CTCGTAGTTCACAATTTCTGGAG
FASN	CATCCAGATAGGCCTCATAGACCTG	CTCCATGAAGTAGGAGTGGAAG
CK18	ATCTTGGTGATGCCTTGGAC	CCTGCTTCTGCTGGCTTAAT
Notch3	GGCTGTGGATTGCCGTCAGTGGA	ATCTGCGTCGCCCTGTGGTGGTGT
TGFBR1	TGGAGAGGAAAGTGGCGGGGAG	GCCTCACGGAACCACGAACG
ACVR1B	TTCCCCCTTGTTGTCCTCCT	TCACACGTGTAGTTGGCCTG


### Zebrafish Xenograft Establishment and Fish Exposure

Wild-type AB zebrafish embryos at 48 hpf were dechorionated prior to cell injection. Approximately 500 CM-DiI (Invitrogen, Carlsbad, CA, United States)-labeled cells were injected into embryonic yolk sacs. Then, the embryos were exposed to ethanol (solvent control) or different bakuchiol concentrations. The exposed embryos were maintained at 28°C for 1 h and then placed in an incubator at 34°C for 7 days. The incubation medium was replaced every 2 days. All procedures with fish were conducted following the License to Conduct Experiments approved by the Government of the Hong Kong SAR Department of Health [Refs. (15–10) in DH/HA&P/8/2/5 Pt.3].

### Lightsheet Microscope Imaging

To determine the percentage of metastasis (number of embryos with tail or head metastasis/number of total embryos), xenotransplanted embryos were examined using a lightsheet microscope (Lightsheet Z.1, ZEISS, Germany) equipped with 5× water lens. Images were taken using Z-stack functions. The results were projected with the maximum projection by using lightsheet software (Zen, ZEISS, Germany).

### Statistical Analysis

Statistical analyses were conducted using SigmaPlot 10.0 and SPSS 13.0. Experiments were conducted in triplicate and data were presented as mean values ± standard deviation. Data were analyzed with Student’s *t*-test and one-way ANOVA. A *p*-value < 0.05 was considered statistically significant.

## Results

### Bakuchiol Decreased Mammosphere Formation in MCF-7 Cells

**FIGURE 1:**
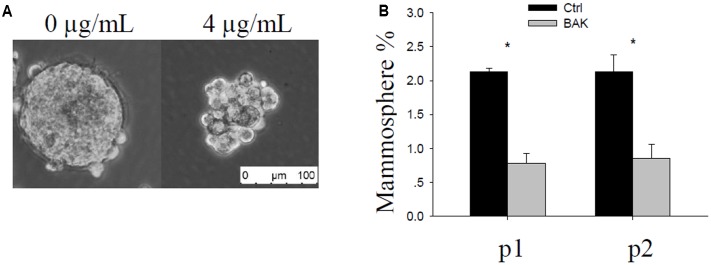
Bakuchiol inhibited mammosphere formation in MCF-7 cells. **(A)** Representative morphology of mammospheres formed in the control and bakuchiol treatment groups. The white bar represents a length of 100 μm. **(B)** Comparison of percentage of mammospheres in the total number of seeded cells. Each condition was performed in triplicate (^∗^*p* < 0.05, *t*-test). Ctrl, control; BAK, bakuchiol.

MCF-7 cells were seeded in ultra-low attachment plates and treated with ethanol (vehicle control) or with 4 or 7 μg/mL bakuchiol for a week to form mammospheres. Mammosphere formation occurred in the control and 4 μg/mL bakuchiol treatment groups (**Figure 1A**) but not in the 7 μg/mL bakuchiol treatment group (data not shown). Mammospheres in control and treatment groups exhibited significant morphological differences: mammospheres in the treatment groups were smaller, looser, and less spherical than those in the control group (**Figure 1A**). The percentages of mammosphere formation (number of mammospheres/number of seeded cells) were downregulated by bakuchiol in both p1 and p2 passages (**Figure 1B**).

### Bakuchiol Inhibited Mammosphere Formation in CD44^+^/CD24^-/low^ BCSCs

**FIGURE 2:**
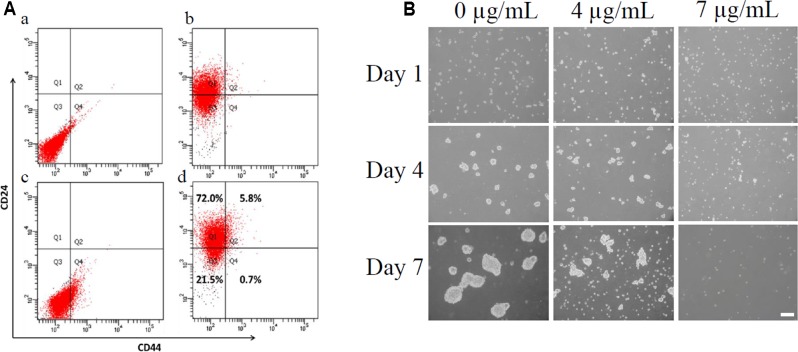
Bakuchiol inhibited mammosphere formation in CD44^+^/CD24^-/low^ BCSCs. **(A)** Analysis of percentage of CD44^+^/CD24^-/low^ population in MCF-7 cells. **(a)** Cells stained with PE-IgG2a and FITC-IgG2b as control. **(b)** Cells stained with PE-CD24. **(c)** Cells stained with FITC-CD44. **(d)** Cells double-stained with PE-CD24 and FITC-CD44. CD44^+^/CD24^-/low^ population was sorted out using a cell-sorting flow cytometer. **(B)** Pictures of mammospheres in CD44^+^/CD24^-/low^ MCF-7 cells in the control and bakuchiol treatment groups on days 1, 4, and 7. The white bar represents a length of 100 μm. The experiments were carried out in triplicates.

The subpopulation of CD44^+^/CD24^-/low^ cells accounted for less than 1% of the total cells (**Figure 2A**). We used this subpopulation of cells for the following mammosphere formation assay. Cells were imaged on days 1, 4, and 7 to monitor mammosphere growth after CD44^+^/CD24^-/low^ cells were treated with different bakuchiol concentrations. The majority of cells were separated on day 1 (**Figure 2B**). On days 4 and 7, control group mammospheres were compact and spherical, and treatment group mammospheres became less compact, fewer in number, and smaller as bakuchiol concentration increased (**Figure 2B**). When cells were exposed to 7 μg/mL bakuchiol, mammospheres appeared only on day 4 but not on day 7 (**Figure 2B**).

### Bakuchiol Reduced ALDH Activity in CD44^+^/CD24^-/low^ BCSCs

**FIGURE 3:**
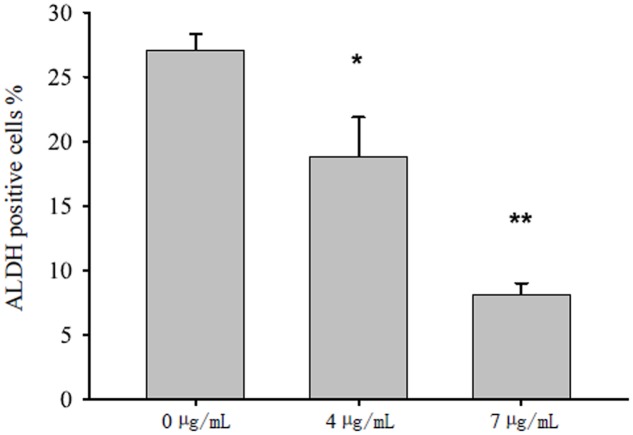
Bakuchiol inhibited ALDH activity in CD44^+^/CD24^-/low^ BCSCs. Cells were exposed to ethanol (vehicle control) or were treated with 4 or 7 μg/mL bakuchiol for 4 days. ALDH activity was detected with a ALDEFLUOR Kit. Each condition was performed in triplicate. Data were presented as mean ± SD (^∗∗∗^*p* < 0.001, ^∗∗^*p* < 0.01, ^∗^*p* < 0.05, one-way ANOVA).

The background ALDH activity varied in different treatments. Thus, we investigated the background ALDH activity of each treatment condition by using DEAB as manufacturer suggested. After 4 days of treatment, bakuchiol significantly reduced the ALDH activity in a dose-dependent manner (**Figure 3**).

### Bakuchiol Induced Apoptosis in CD44^+^/CD24^-/low^ BCSCs

**FIGURE 4:**
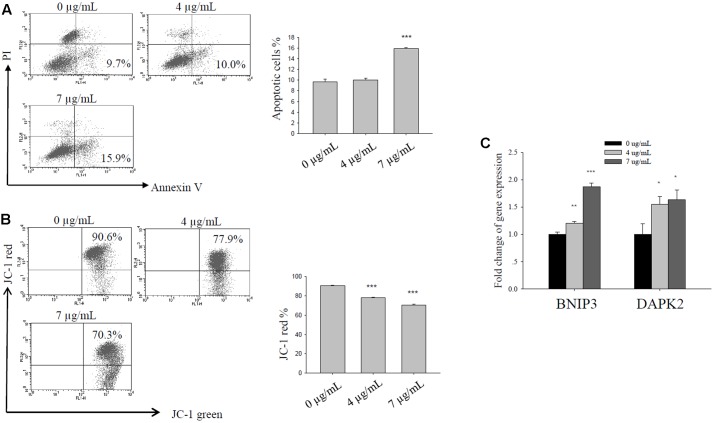
Bakuchiol induced apoptosis in CD44^+^/CD24^-/low^ BCSCs. Cells were exposed to ethanol (vehicle control), 4, or 7 μg/mL bakuchiol for 4 days. **(A)** Analysis of bakuchiol-induced early apoptosis in CD44^+^/CD24^-/low^ cells with Annexin V/PI staining. **(B)** JC-1 staining for the analysis of mitochondrial membrane potential in CD44^+^/CD24^-/low^ cells. **(C)** mRNA expression levels of BNIP3 and DAPK2 in CD44^+^/CD24^-/low^ cells. GAPDH was used as an internal reference. The experiments were carried out in triplicates (^∗^*p* < 0.05, ^∗∗^*p* < 0.01, ^∗∗∗^*p* < 0.001, one-way ANOVA).

We exposed CD44^+^/CD24^-/low^ cells to ethanol (vehicle control) or to 4 or 7 μg/mL bakuchiol for 4 days. We then analyzed cell apoptosis using Annexin V/PI staining and mitochondrial membrane potential using JC-1 staining. As bakuchiol concentration increased, the percentage of early apoptotic cells increased (**Figure 4A**) and mitochondrial membrane potential decreased (**Figure 4B**) in CD44^+^/CD24^-/low^ cells. The mRNA levels of pro-apoptotic genes, BNIP3 and DAPK2 were significantly upregulated in cells after bakuchiol treatment (**Figure 4C**).

### Bakuchiol Induced Oxidative Stress in CD44^+^/CD24^-/low^ BCSCs

**FIGURE 5:**
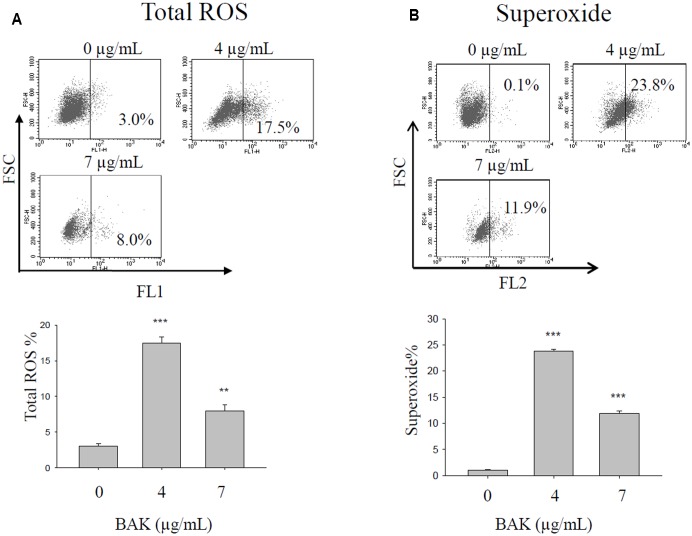
Bakuchiol treatment changed ROS production in CD44^+^/CD24^-/low^ BCSCs. Cells were exposed to ethanol (vehicle control) or were treated with 4 or 7 μg/mL bakuchiol for 4 days. Total ROS **(A)** and superoxide **(B)** production was determined with a ROS/Superoxide detection kit. Each condition was performed in triplicate. Data were presented as mean ± SD (^∗∗∗^*p* < 0.001, ^∗∗^*p* < 0.01, ^∗^*p* < 0.05, one-way ANOVA). BAK, bakuchiol.

After 4 days of bakuchiol treatment, staining with ROS detection dye revealed that bakuchiol induced total ROS production (**Figure 5A**) and superoxide production (**Figure 5B**). Although total ROS production and superoxide production were upregulated in both treatment groups, total ROS production and superoxide production in the 7 μg/mL treatment group were lower than that in 4 μg/mL group (**Figures 5A,B**).

### Bakuchiol Affected the Lipogenesis of CD44^+^/CD24^-/low^ BCSCs

**FIGURE 6:**
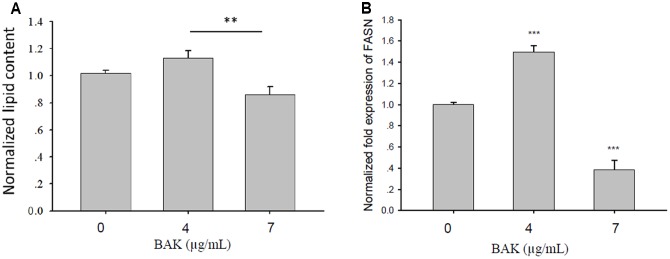
Bakuchiol affected lipogenesis of CD44^+^/CD24^-/low^ BCSCs. Cells were exposed to ethanol (vehicle control) or treated with 4 or 7 μg/mL bakuchiol for 4 days. **(A)** Intracellular lipid content was quantified using AdipoRed Assay Reagent. Relative fold changes of lipid content were normalized to the level of control (arbitrarily set to 1). Data were presented as mean ± SD. **(B)** mRNA expression levels of FASN in CD44^+^/CD24^-/low^ cells. GAPDH was used as an internal reference. The experiments were carried out in triplicates (^∗∗∗^*p* < 0.001, ^∗∗^*p* < 0.01, ^∗^*p* < 0.05, one-way ANOVA). BAK, bakuchiol.

We analyzed lipid content and FASN mRNA expression levels in CD44^+^/CD24^-/low^ cells that were exposed to different bakuchiol concentrations for 4 days. AdipoRed staining revealed that lipid content increased in cells that were exposed to 4 μg/mL bakuchiol but decreased in cells that were exposed to 7 μg/mL bakuchiol (**Figure 6A**). Lipid contents in the treatment and control groups were not significantly different, whereas those in the two treatment groups were significantly different (**Figure 6A**). FASN mRNA level was elevated in the 4 μg/mL bakuchiol treatment group and downregulated in the 7 μg/mL bakuchiol treatment group (**Figure 6B**). The trend of upregulation at 4 μg/mL and downregulation at 7 μg/mL is consistent with that for lipid content.

### Bakuchiol Inhibited MCF-7 Cell Metastasis in Zebrafish Embryos

**FIGURE 7:**
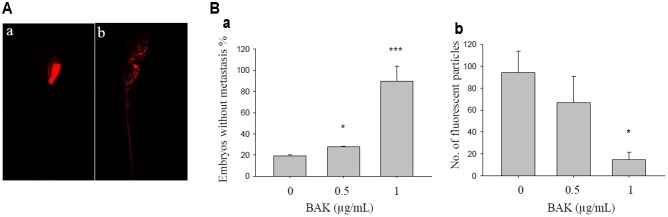
Bakuchiol inhibited *in vivo* cell metastasis in zebrafish. **(A)** Representative images of zebrafish embryo **(a)** without and **(b)** with cell metastasis. **(B)** Approximately 500 unsorted MCF-7 cells were injected into the yolk sacs of 48 hpf zebrafish embryos. Then, 40 embryos were treated with ethanol (solvent control) or with 0.5 or 1 μg/mL of bakuchiol for 7 days. Each condition was performed in triplicate. Statistical analysis of **(a)** the percentages of embryos without cell metastasis in control and treatment groups, and of **(b)** the numbers of fluorescent particles in control and treatment groups (^∗∗∗^*p* < 0.001, ^∗∗^*p* < 0.01, ^∗^*p* < 0.05, one-way ANOVA). BAK, bakuchiol.

Cell metastasis was observed on day 7 after cell injection in 48-hpf zebrafish embryos that were exposed to 0, 0.5, and 1 μg/mL bakuchiol. The representative images of embryos with and without cell metastasis are shown in **Figure 7A**. The percentages of embryos without metastasis increased in a dose-dependent manner, and the numbers of fluorescent particles decreased in a dose-dependent manner (**Figure 7B**). Mortality in the control and treatment groups was not significantly different, as is consistent with our previous result (Li et al., 2016).

### Bakuchiol Altered Metastasis-Related Gene Expression in CD44^+^/CD24^-/low^ BCSCs

**FIGURE 8:**
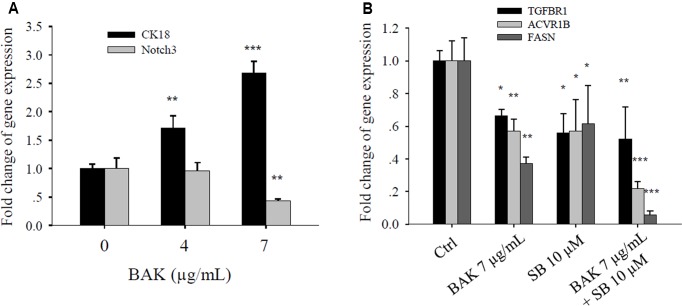
Bakuchiol affected the metastasis-related gene expression in CD44^+^/CD24^-/low^ BCSCs. **(A)** Cells were exposed to ethanol (vehicle control) or were treated with 4 or 7 μg/mL bakuchiol for 4 days. The mRNA expression levels of CK18 and Notch3 were quantified by real-time PCR. **(B)** Cells were treated with ethanol (Ctrl) or with 7 μg/mL bakuchiol (BAK) or/and 10 μM SB-431542 (SB) for 4 days. The mRNA expression levels of TGFBR1, ACVR1B, and FASN were quantified by real-time PCR. GAPDH was used as an internal reference. The experiments were carried out in triplicates (^∗^*p* < 0.05, ^∗∗^*p* < 0.01, ^∗∗∗^*p* < 0.001, one-way ANOVA). BAK, bakuchiol.

Considering that bakuchiol inhibits cell metastasis in zebrafish embryos, we investigated how bakuchiol affected the expression levels of metastasis-related genes in CD44^+^/CD24^-/low^ cells. Treatment with different concentrations of bakuchiol upregulated CK18 and downregulated Notch3 in a dose-dependent manner (**Figure 8A**). Genes in the transforming growth factor-β (TGF-β) signaling pathway, such as TGFBR1 and ACVR1B, decreased upon exposure to bakuchiol. When cells were co-treated with the TGF-β signaling pathway inhibitor SB-431542, the expression levels of TGFBR1 and ACVR1B further decreased (**Figure 8B**). FASN expression exhibited a similar trend (**Figure 8B**).

## Discussion

In our previous study, we found that high-dose bakuchiol inhibited MCF-7 cells in a dose-dependent manner; and we investigated the mechanisms of bakuchiol-induced apoptosis and cell cycle arrest with the doses of 4 and 7 μg/mL in that study (Li et al., 2016). We adopted the same doses in current study to investigate if bakuchiol can target BCSCs. Mammosphere formation has been developed as an *in vitro* assay to confirm the self-renewal potential of BCSCs. The results of the present study revealed that bakuchiol inhibits self-renew of BCSCs. The ALDHs are a group of NAD(P)^+^-dependent enzymes that are involved in oxidizing aldehydes. The expression of ALDH is enriched in cancer stem cells (Ginestier et al., 2007; Kakarala and Wicha, 2007). We showed that bakuchiol inhibited the ALDH activity in BCSCs, which further proved its anti-BCSCs effect. Moreover, bakuchiol induces the apoptosis of BCSCs, and inhibits the metastasis of breast cancer cells in zebrafish embryos. Thus, we examined the possible signaling pathways, including ROS production, lipogenic, pro-apoptotic, Notch, cytoskeletal, and TGF-β signaling pathways, which underlie these effects.

Reactive oxygen species can be categorized as free oxygen radicals (such as superoxide and nitric oxide) and non-radical ROS (such as hydrogen peroxide, singlet oxygen, and ozone/trioxygen) (Liou and Storz, 2010). With threshold limits, ROS contribute to cancer cell homeostasis and growth. However, aberrant ROS that exceeds the threshold can elicit cancer cell apoptosis through both extrinsic (receptor-mediated) and intrinsic (mitochondrial-mediated) apoptotic pathways (Ryter et al., 2007; Liou and Storz, 2010). BNIP3 is a pro-apoptotic BH3-only protein, functioning as a mitochondrial stress sensor. Activation of BNIP3 results in loss of mitochondrial membrane potential, generation of ROS, and induction of apoptosis (Chinnadurai et al., 2008). However, the role of ROS in cancer metastasis remains controversial. Piskounova et al. (2015) reported that ROS limit distant metastasis and that only cells with increased antioxidant capacity can metastasize. Meanwhile, several other studies have reported contrasting results, as reviewed by Peiris-Pagès et al. (2015). In the present study, bakuchiol induced total ROS and superoxide production in CD44^+^/CD24^-/low^ BCSCs, implicating the potential of bakuchiol as a ROS-inducing agent for chemotherapy.

FASN is a key lipogenic enzyme that catalyzes the terminal steps in the biogenesis of fatty acids. FASN is lowly expressed or undetectable in most tissues but is overexpressed in a wide range of epithelial tumors, including breast cancer, because rapidly growing cancer cells require lipids for membrane synthesis and energy supply (Kuhajda, 2000). The inhibition of FASN can induce apoptosis in breast cancer cells through upregulating pro-apoptotic genes BNIP3, DAPK2, and TRAIL (Bandyopadhyay et al., 2006). Several phytoestrogens, such as resveratrol and genistein, suppress BCSCs by inhibiting FASN and mammary lipogenesis (Pandey et al., 2011; Montales et al., 2013). The FASN inhibitor attenuates the metastasis of different tumors, such as melanomas, breast cancer, oral cancer, and colorectal cancer (Seguin et al., 2012; Zaytseva et al., 2012, 2015; Agostini et al., 2014; Singh et al., 2015). Pharmacological limitations and side effects of current FASN inhibitors prevent their development as systemic drugs (Flavin et al., 2010). However, natural product-derived FASN inhibitors, such as bakuchiol, may provide new therapeutic moieties for breast cancer patient care. In the current study, treatment with 7 μg/mL bakuchiol decreased lipid content and repressed FASN expression in CD44^+^/CD24^-/low^ BCSCs, suggesting that bakuchiol may induce apoptosis in BCSCs and inhibit *in vivo* metastasis by inhibiting lipogenesis. However, treatment with 4 μg/mL bakuchiol increased lipid content and FASN expression, and induced a high level of oxidative stress. In our previous study (Li et al., 2016), we found that bakuchiol exerted a biphasic effect on the growth of MCF-7 cells – stimulating cellular proliferation at low concentrations because of the ER agonist effect, and inhibiting cell proliferation at high concentrations because of the ER antagonist effect. The concentration of 4 μg/mL is around the biphasic border, which may explain the differences in oxidative stress and lipogenesis between 4 and 7 μg/mL such as a narrow window. Thus, further clinical experiments should be carefully designed.

Transforming growth factor-β induces the expression of cell surface markers associated with cancer stem cells (Mani et al., 2008). By regulating EMT-related and extravasation-related genes, TGF-β induces tumor invasion and metastasis in numerous cancer models (reviewed by Sheen et al., 2013; Cantelli et al., 2017). Cytokeratins are the major component of the epithelial cytoskeleton, which provide mechanical stability to tissues. CK18 is downregulated in metastatic breast cancer (Hedenfalk et al., 2001; Zajchowski et al., 2001; Woelfle et al., 2004). The EMT–FASN positive loop may contribute to the metastatic potential of breast and lung cancers (Hung et al., 2011; Li et al., 2014; Yang et al., 2016). In the current study, we showed that bakuchiol inhibited the mRNA expression of TGF-β receptors (TGFBR1 and ACVR1B), CK18, and FASN. The co-treatment of bakuchiol with the TGF-β receptor inhibitor SB-431542 further suppressed the expression levels of TGFBR1, ACVR1B, and FASN, thus implicating that TGF-β may be involved in the bakuchiol-induced inhibition of metastasis.

Notch signaling promotes the self-renewal capacity of mammospheres. Dontu et al. (2004) have reported a 10-fold increase in secondary mammosphere formation upon the addition of a Notch-activating delta-Serrate-Lag2 peptide. Notch inhibition decreases ductal carcinoma *in situ*-derived mammosphere production (Farnie and Clarke, 2007). Notch 3 is involved in mammosphere formation (Sansone et al., 2007). Moreover, positive feedback between Notch 3 and TGF-β contributes to the metastasis of breast and lung cancers (Zhang et al., 2010; Liu et al., 2014). In the present study, we found that bakuchiol repressed Notch 3 expression, possibly exerting an inhibitory effect on the self-renewal capacity of BCSCs and metastasis of breast cancer cells.

Zebrafish xenograft has increasingly become a desirable tool for cancer metastasis study (Tat et al., 2013; Teng et al., 2013; Heilmann et al., 2015; Brown et al., 2017). In current study, we found that bakuchiol inhibited breast cancer cell metastasis in zebrafish embryos. Although we have investigated several pathways that may be related to the anti-metastasis effect with *in vitro* experiments, including FASN, TGF-β, and Notch. The doses we used for *in vivo* metastasis experiment were lower than that for *in vitro* experiments. According to our previous study (Li et al., 2016) and current study, low-dose bakuchiol induced *in vitro* MCF-7 cell growth, but also inhibited *in vivo* cell growth and cell metastasis. Thus, the mechanisms described at higher doses may not be utilized to explain the *in vivo* finding. To further investigate if these signaling pathways are involved in the *in vivo* anti-metastasis effects, cells with overexpression of FASN, TGFBR1, ACVR1B, and Notch3 should be injected to see if the bakuchiol-induced anti-metastasis effects can be rescued.

## Conclusion

Our results showed that bakuchiol targets BCSCs and inhibits breast cancer cell metastasis in zebrafish embryos. These findings suggest the potential of bakuchiol in breast cancer treatment. However, further experiments should be carried out to overcome the limitations of current study.

## Author Contributions

LL was involved in the project design, carried out most of the experiments, and drafted the manuscript. CL helped with the zebrafish xenograft establishment and imaging. XC participated in fish maintenance and exposure. SX and SH helped with the ALDH activity experiment. SC contributed substantially to the experimental design, manuscript preparation, and submission. All authors read and approved the final manuscript.

## Conflict of Interest Statement

SC served on the Scientific Advisory Board of the Company Vitargent Biotechnology Limited. XC was employed by the company Vitargent Biotechnology Limited. The other authors declare that the research was conducted in the absence of any commercial or financial relationships that could be construed as a potential conflict of interest.

## Footnotes

1 http://www.cancer.net/cancer-types/breast-cancer/statistics
